# Identification of the potato (*Solanum tuberosum* L.) P-type ATPase gene family and investigating the role of *PHA2* in response to Pep13

**DOI:** 10.3389/fpls.2024.1353024

**Published:** 2024-06-06

**Authors:** Feng Zhang, Anping Yuan, Zongyue Nie, Moli Chu, Yanlin An

**Affiliations:** ^1^ Department of Food Science and Engineering, Moutai Institute, Renhuai, Guizhou, China; ^2^ Agriculture Science Institute of Bijie, Bijie, Guizhou, China; ^3^ Anhui Provincial Key Laboratory of the Conservation and Exploitation of Biological Resources/College of Life Sciences, Anhui Normal University, Wuhu, Anhui, China

**Keywords:** P-type ATPase, potato, PM H + -ATPase, Pep13, gene family

## Abstract

P-type ATPase family members play important roles in plant growth and development and are involved in plant resistance to various biotic and abiotic factors. Extensive studies have been conducted on the P-type ATPase gene families in *Arabidopsis thaliana* and rice but our understanding in potato remains relatively limited. Therefore, this study aimed to screen and analyze 48 P-type ATPase genes from the potato (*Solanum tuberosum* L.) genome database at the genome-wide level. Potato P-type ATPase genes were categorized into five subgroups based on the phylogenetic classification of the reported species. Additionally, several bioinformatic analyses, including gene structure analysis, chromosomal position analysis, and identification of conserved motifs and promoter cis-acting elements, were performed. Interestingly, the plasma membrane H^+^-ATPase (PM H^+^-ATPase) genes of one of the P3 subgroups showed differential expression in different tissues of potato. Specifically, *PHA2*, *PHA3*, and *PHA7* were highly expressed in the roots, whereas *PHA8* was expressed in potatoes only under stress. Furthermore, the small peptide Pep13 inhibited the expression of *PHA1*, *PHA2*, *PHA3*, and *PHA7* in potato roots. Transgenic plants heterologously overexpressing *PHA2* displayed a growth phenotype sensitive to Pep13 compared with wild-type plants. Further analysis revealed that reducing potato PM H^+^-ATPase enzyme activity enhanced resistance to Pep13, indicating the involvement of PM H^+^-ATPase in the physiological process of potato late blight and the enhancement of plant disease resistance. This study confirms the critical role of potato *PHA2* in resistance to Pep13.

## 1 Introduction

P-ATPase genes belong to a protein family that is widely present in plant cell membranes (Pedersen et al., 2012; Zhang et al., 2020). Their main function is to facilitate ion transport across membranes via ATP hydrolysis. This process is crucial for maintaining intracellular ion homeostasis and plays a significant role in important physiological processes such as plant growth and development (Pedersen et al., 2012). The P-type ATPase superfamily can be classified into five major subfamilies based on the sequence properties and functions of the P-type ATPase genes (Palmgren and Nissen, 2011; Pedersen et al., 2012). The P1 subfamily is dominated by the P1A branch, which has more members than the P1B branch (Axelsen and Palmgren, 1998). The P2 subfamily comprises four branches: P2A, P2B, P2C, and P2D. Previous research has suggested that the P2A and P2B branches are primarily responsible for transporting Ca^2+^ (Palmgren and Nissen, 2011). The P2C branch is believed to be involved in the transport of Na^+^/K^+^ and H^+^/K^+^ ions. The P2D branch represents a minority of the proteins within the P2 subfamily (Palmgren and Nissen, 2011). In the P3 subfamily, P3A functions as a P-type H^+^-ATPase, whereas P3B is associated with Mg^2+^-ATPases in certain bacterial species (Axelsen and Palmgren, 1998). The P4 subfamily is involved in lipid transport. The functional characteristics of the P5 subfamily have not yet been fully elucidated (Baxter et al., 2003). A total of 46 P-ATPase genes have been identified in *Arabidopsis* (Palmgren, 2001) and 43 in rice (Baxter et al., 2003). *Arabidopsis* and rice exhibit a high abundance of genes in five subfamilies: P1B, P2A, P2B, P3A, and P4. However, the P5 subfamily is characterized by a scarcity of genes and the P2C, P2D, P1A, and P3B subfamilies lack identified genes (Baxter et al., 2003). P-type H^+^-ATPases belong to the P3A subfamily and are found in fungi and plants (Axelsen and Palmgren, 1998). These enzymes have a common structure consisting of ten transmembrane helices and three cell membrane structural domains: the N-terminal, catalytic, and C-terminal domains (Pedersen et al., 2007). P-type H^+^-ATPase uses the energy stored in ATP to transfer protons during the catalytic cycle. In most plants, these enzymes are localized in the plasma membrane; therefore, they are referred to as plasma membrane H^+^-ATPases (PM H^+^-ATPases).

PM H^+^-ATPase is encoded by multiple genes and there is significant variation in the quantity and type of this protein among different species (Saijo et al., 2018). This protein exhibits a certain degree of tissue and organ specificity and may partially overlap in function. Taking *Arabidopsis thaliana* as an example, 12 functional members of the PM H^+^-ATPase family (*AtAHA1–12*) have been identified (Axelsen and Palmgren, 2001). Genetic analysis has revealed that *AtAHA1* plays a role in steroid signaling, *AtAHA2* is involved in iron transport, *AtAHA3* participates in pollen development, *AtAHA4* is associated with salt stress, and *AtAHA10* is involved in vacuole biogenesis (Vitart et al., 2001; Baxter et al., 2005; Haruta et al., 2010; Hoffmann et al., 2019). In addition, eight *AtAHAs* are expressed in green leaves, eight in roots, and three in all tissues (Yuan et al., 2017). In maize (*Zea mays* L.), four similar H^+^-ATPases (*ZmHA1–4*) have been identified (Frías et al., 1996). In tobacco (*NpHA1–9*), the proton pump gene *NpHA1* is only expressed in the root epidermis, *NpHA5* in transport cells, and *NpHA6* and *NpHA9* in the leaf trichomes and cortex, respectively (Oufattole et al., 2000). In rice (*Oryza sativa*), this family consists of 10 members that are divided into five subgroups called PM H^+^-ATPase 1 (*OSA1*) through *OSA10* (Toda et al., 2016). Until now, in potato *(Solanum tuberosum* L.), seven genes encoding plasma membrane H^+^-ATPases have been identified, designated *PHA1*–*PHA7* (Stritzler et al., 2017). Interestingly, studies have identified eight transcriptionally active plasma membrane H^+^-ATPase genes, named *SlHA1–8*, in tomato, which belongs to the same family as potato, *Solanaceae*. Among these genes, *SlHA8* is strongly and exclusively expressed in roots infected with mycorrhizal fungi (Liu et al., 2016, 2020).

Alkalinization of extracellular pH has long been regarded as a hallmark of plant immune responses, especially in the study of pathogen-associated molecular pattern (PAMP)-triggered immunity (PTI) (Liu et al., 2022). Plant immunologists have successfully identified the active forms of many important PAMP immune molecules, such as flg22 and Pep1, using extracellular alkalinization as an indicator (Felix et al., 1999; Jeworutzki et al., 2010). The MAMP peptide, flg22, derived from bacterial flagellin proteins, induces extracellular alkalinization by altering the phosphorylation status of *AHA1/AHA2*. Specifically, flg22 decreased the phosphorylation of Thr881 and Thr947, while increasing the phosphorylation of Ser899. These phosphorylation and dephosphorylation events reduce the activity of PM H^+^-ATPase (Nühse et al., 2007). A recent study revealed the mechanism by which cell-surface peptide-receptor complexes act as extracellular pH sensors that regulate plant growth and immunity (Liu et al., 2022). Through *in situ* staining with HPTS, researchers have observed that the extracellular environment in the root apical meristem cells of plants is relatively acidic. Treatment of plants with the immune peptide Pep1 or PAMPs rapidly increases the extracellular pH in the root apical meristem, indicating that immune responses cause extracellular alkalinization in this tissue. Furthermore, the immune response elicited by Pep1 significantly inhibited root apical meristem growth. Further experiments showed that the immune response induced by Pep1 inhibited the signaling pathway of the peptide hormone RGF1, which promotes root stem cell growth and reduces the expression of the key transcription factor PLT1/2 involved in root apical growth and development. Interestingly, the region of Pep1-induced extracellular alkalinization in the root apical meristem highly coincides with the expression regions of RGF1 and PLT1/2. RGF1 is a secreted peptide-signaling molecule that interacts extracellularly with its receptor (Liu et al., 2022).

Some fungi selectively regulate the activity of plant PM H^+^-ATPase. For example, a fungus secretes thiadiazole acid, which inhibits the activity of PM H^+^-ATPase through its C-terminal domain, leading to cell death (Bjørk et al., 2020). PM H^+^-ATPases play an important role in the interaction between plants and fungi. Fungi of the genus *Trichoderma* benefit plant growth (Yedidia et al., 2001; Contreras-Cornejo et al., 2014). Although the specific mechanism is not clear, the activity of plasma membrane H^+^-ATPase is regulated by *Trichoderma* (López-Coria et al., 2016), suggesting that the activation of plasma membrane H^+^-ATPase may be one of the beneficial mechanisms of *Trichoderma asperellum* for plants. Studies have also found that the addition of inducers leads to rapid alkalization of the growth medium, which may be achieved by inhibiting the activity of plasma membrane H^+^-ATPase (Saijo et al., 2018). *Fusarium oxysporum* can induce rapid acidification of the apoplast, thereby regulating cell wall structure and root growth by reducing cellulose synthesis. Infection with the fungus *Fusarium oxysporum* also leads to Thr947 phosphorylation and it is worth noting that treatment with the inducer of the fungus *Fusarium oxysporum* produces the same effect (Kesten et al., 2019). However, a class of substances called RALF-like peptides secreted by the fungus *Fusarium oxysporum* has the opposite effect. These F-RALFs inhibit the activity of the plasma membrane H^+^-ATPase through an iron-dependent pathway, thereby triggering apoplastic alkalinization (Masachis et al., 2016).

As a globally important crop, potatoes are constantly threatened by various pathogens, especially late blight caused by the oomycete *Phytophthora infestans* (Trout et al., 1997; Lin et al., 2023). This disease has a severe impact on potato yield, sometimes leading to complete crop failure and posing significant risks to farming and related industries. Pep13 is an oligopeptide derived from extracellular transglutaminase produced by *Phytophthora* species and it is a PAMP that triggers a variety of immune responses (Brunner et al., 2002). Recent studies have successfully identified the immune receptor PERU in potatoes, which recognizes the immunogenic peptide Pep13. When PERU binds to Pep13, it induces immune responses in potato plants, including cell death, ROS bursts, and ethylene production (Torres Ascurra et al., 2023). However, the role of the plasma membrane H^+^-ATPase gene family in potatoes during the late blight caused by *Phytophthora infestans* remains unclear.

In the present study, a bioinformatics approach was employed to comprehensively identify and analyze the P-type ATPase gene family in potatoes. The sequence characteristics, chromosomal localization, gene structure, evolutionary relationships, and promoter elements of these genes were systematically analyzed, particularly focusing on the expression patterns of P-type H^+^-ATPase gene family members in the roots, stems, and leaves. Additionally, we treated potato roots, stems, and leaves with Pep13 and analyzed its differential expression in different tissues. Through these investigations, a comprehensive understanding of the members of the potato P-type ATPase gene family and their functional roles in late blight occurrence was achieved, providing important references for further research.

## 2 Materials and methods

### 2.1 Selection of plant materials and optimization of growth conditions

*Arabidopsis thaliana* Columbia-0 (Col-0) seeds were obtained from Nanjing University. To promote growth, the seeds were sterilized using 10% sodium hypochlorite for 4 min, rinsed four times with double-distilled water, and planted on solidified Murashige and Skoog (MS) or 1/4 MS medium with 1% sucrose (Aldrich-Sigma) and 1% agar (Aldrich-Sigma). The growth phenotype of Pep13 in *Arabidopsis* was assessed as previously described (Jing et al., 2019). The plates were initially incubated in darkness at 4°C for 2 days and then positioned vertically. On day 10 after germination, primary root lengths were measured using ImageJ software (http://rsb.info.nih.gov/ij/) and the biomass was weighed.

The potato variety Unk ‘Atlantic’ was selected to investigate the effects of Pep13. This variety was obtained from the Institute of Vegetables and Flowers of the Chinese Academy of Agricultural Sciences. After germination at 22°C, the pre-elite seeds were then transplanted into a solution containing 1/4 potato Murashige and Skoog (MS) basal salts with vitamins (Coolaber). The initial pH of this solution was 5.5. The cultivation of the potatoes took place in growth chambers set at 25°C, with a 12-h light/12-h dark photoperiod.

### 2.2 Identification and analysis of P-type ATPase gene family

The potato P-type ATPase gene was identified using potato gene, transcript, and protein sequences downloaded from the Phytozome website (*Solanum tuberosum* v6.1, Phytozome genome ID: 686, and NCBI taxonomy ID: 4113) (Pham et al., 2020). The P-type ATPase domain (sequence numbers: PF00690, PF00122, and PF13246) was used as a reference (Zhang et al., 2020) to search for P-type ATPase in the Potato Protein Sequence Database using the Hidden Markov Model (HMM). Sequences containing the structural domain of the P-type ATPase were identified and species-specific models were created. Candidate potato P-type ATPase gene sequences were further confirmed using BLAST (1e-10) to ensure their membership in the P-type ATPase gene family. Redundant sequences were removed and the resulting P-type ATPase protein sequences were submitted to the Pfam (http://pfam.janelia.org/), NCBI Conserved Domain (http://www.ncbi.nlm.nih.gov/Structure/cdd/wrpsb.cgi), and SMART (http://smart.em-bl-heidelberg.De) databases for additional confirmation of the P-type ATPase motifs. Finally, erroneous and repetitive sequences were eliminated to identify the members of the P-type ATPase gene family. Secondary and tertiary structures of the proteins were predicted using the SOPMA database (https://npsa-prabi.ibcp.fr/cgi-bin/npsa_automat.pl?page=/NPSA/npsa_server.html) and Phyre2 software (http://www.sbg.bio.ic.ac.uk/phyre2/html/page.cgi?id=index), respectively (Gasteiger, 2005; Kelley et al., 2015).

### 2.3 Phylogenetic analysis and motif identification

Multiple sequence comparisons were performed using the ClustalW tool in MEGA 7 software on the sequences of the P-type PM H^+^-ATPase proteins of *Solanum tuberosum* (PHA), *Solanum lycopersicum* (HA), *Arabidopsis thaliana* (AHA), *Oryza sativa* (OSA), *Helianthus annuus* (HHA), and *Nicotiana plumbaginifolia* (PMA) (Stritzler et al., 2017; Xu et al., 2020). Subsequently, neighbor-joining (NJ) phylogenetic trees were constructed based on the Poisson model, and the robustness of the dendrograms was evaluated by setting bootstrap values for 1000 times. To gain a deeper understanding of the potential properties of these protein sequences, themes in the sequences were further predicted using MEME software (http://meme-suite.org/) (Lu et al., 2021).

### 2.4 Chromosomal distribution, gene duplication, and synteny analysis of P-type ATPase gene family

The chromosomal distribution of gene family members was determined using the GFF file of the reference genome v6.1 and the distribution was visualized using the MG2C online web service (http://mg2c.iask.in/mg2c_v2.1/). Gene duplication and collinearity analyses were conducted and visualized using the multicollinearity scanning function module in the TBtools software, as described in our previous study (An et al., 2022).

### 2.5 Reverse transcription-quantitative polymerase chain reaction techniques for gene expression analysis

Total RNA was extracted from potato and *Arabidopsis* roots using the TRIzol reagent (Invitrogen). The extracted RNA was used to synthesize poly(dT)-complementary DNA using M-MLV reverse transcriptase (Promega). For reverse transcription-quantitative polymerase chain reaction (RT-qPCR), the SYBR Green I Master Kit (Roche Diagnostics) was used on a CFX Connect Real-Time System (Bio-Rad), following the manufacturer’s instructions. RT-qPCR primers for eight members of the PM H^+^-ATPase gene family were designed using Primer Premier 5.0. *ACTIN* (Soltu.DM.03G011750.2) was used as an internal reference gene (Zhang et al., 2022). The primers used are listed in **Supplementary Table S1**. Each sample was replicated three times and differential expression was determined using the 2 [- Delta C(T)] method (Livak and Schmittgen, 2001).

### 2.6 Synthesis of Pep13 small peptides

The Pep13 small peptide (sequence VWNQPVRGFKVYE) utilized in this study was synthesized by Sangon Biotech (Shanghai, China). To meet the experimental requirements, the peptide was dissolved in water to prepare a stock solution at a concentration of 1 mM.

### 2.7 Cloning of the full-length coding sequence of the PHA2 gene

The Atlantic root was chosen as the source for RNA extraction, and total RNA was isolated using an extraction kit from Tiangen Biochemical Technology Co., Ltd. (Beijing, China). Reverse transcription was used to synthesize cDNA. PCR amplification was conducted using the cDNA as a template with the primers *PHA2*-F1 and *PHA2*-R1 (**Supplementary Table S1**). The amplified products were separated by 1% agarose gel electrophoresis. The concentration of the *PHA2* fragment was determined after recovery using a DNA Gel Extraction Kit. The recovered products were polyadenylated using the Taq enzyme and ligated into the pMD 19-T vector. The ligated vector was transformed into *E.coli* DH 5α competent cells and the cells were cultured on a screening medium containing antibiotics. Finally, monoclonal colonies were selected for PCR and their accuracy was confirmed by sequencing.

### 2.8 Over-expression vector construction and *Arabidopsis* transformation

To create expression vectors for the *PHA2 t*ransgenic expression vector, the *PHA2* coding sequence (CDS) was amplified from cDNA obtained from the Atlantic. For *Arabidopsis* transformation, the *PHA2* sequence was cloned into the SpeI/NheI sites of the plant expression vector pCAMBIA1302, resulting in the generation of the plant overexpression vector pCAMBIA1302–35S-*PHA2*. The resulting vector was then transferred into *Agrobacterium tumefaciens* strain GV3101, and *Arabidopsis* Col-0 plants were transformed using the floral dip method (Clough and Bent, 1998). The seeds were sterilized and plated on a solid 1/2 MS medium containing 35 μg mL^–1^ of kanamycin. After 4 days of cultivation at 4°C, the seeds were transferred to a growth chamber set to a light cycle of 16 h of light and 8 h of dark at 25°C for 8 days. The plants were then transplanted into the soil. To screen for positive plants, specific primers (**Supplementary Table S1**) were designed for the PCR identification of transgenic *Arabidopsis*. Three heterologously overexpressing homozygous T3 transgenic lines (OE strains) were obtained for each gene and the best OE-expressing strain (OE–*PHA2-#5*) was selected for subsequent Pep13 experiments (**Supplementary Figure S1**).

### 2.9 Measurements of PM H^+^-ATPase activity

To investigate the effects of Pep13 treatment on Atlantic roots, a 1/6 MS solution without sucrose (pH 5.8) was used as the growth medium. Three-week-old Atlantic roots were transferred to medium with or without 20 nM Pep13. After 3 d of treatment, the roots were collected for further analysis. Plasma membrane vesicles were prepared from the collected roots following a previously described protocol (Shen et al., 2005). To determine the feasibility of the assay, 10 µM vanadate (VA) was used. The vanadate-sensitive ATPase accounted for 85% of the total activity in the plasma membrane fraction. The activity of the PM H^+^-ATPase was measured at a wavelength of 700 nm using a U-2910 spectrophotometer (HITACHI, Tokyo, Japan).

### 2.10 Statistical analysis

Statistical analyses were conducted using Student’s t-test (P<0.05) on at least three independent replicates for all experiments.

## 3 Results

### 3.1 Identification and analysis of members of the P-type ATPase gene family

**Figure 1:**
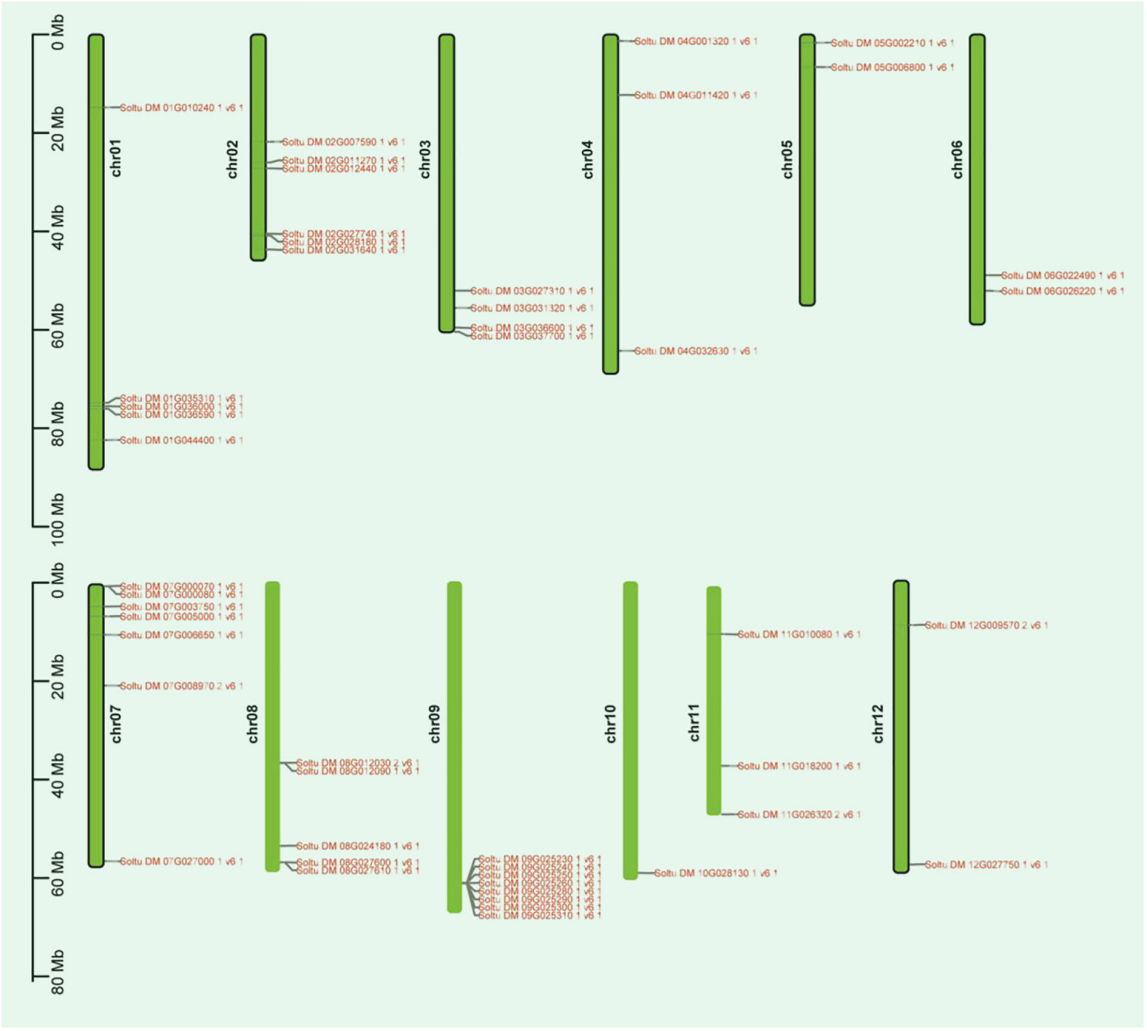
Distribution of the P-type ATPase gene family on potato chromosomes is illustrated in the diagram. The length (Mb) of the chromosomes is represented by the scale bar on the left. P-type ATPase gene family are indicated on both sides of their respective chromosomes.

This study successfully identified 48 members of the potato P-type ATPase gene family from the potato genome database using Biastp online comparison and an HMMER search. This is similar to *Arabidopsis* and rice, which have 46 and 43 P-type ATPase genes, respectively (Palmgren, 2001; Baxter et al., 2003). To better understand the chromosomal distribution of P-type ATPase genes, we used the potato genome database to create a chromosomal localization map (**Figure 1**). The findings revealed that these genes were unevenly distributed across the 12 chromosomes. Chromosome chr09 exhibited the highest number of markers, encompassing eight genes. In contrast, chromosome chr10 had fewer markers, with only one gene identified as Soltu.DM.10G028130.1. The 48 identified PTAP protein sequences were used to construct a phylogenetic tree to determine the subfamily classification of potato P-type ATPase genes. The results showed that the potato P-type ATPase genes could be categorized into five subfamilies (**Supplementary Figure S2**), which is consistent with findings in other plants (Palmgren and Nissen, 2011; Pedersen et al., 2012). Among these, the number of genes in the P4 subfamily was low, whereas the number of genes in the other subfamilies was high. Interestingly, the eight PM H^+^-ATPase members of the P3A subfamily have relatively similar evolutionary homology.

### 3.2 Gene duplication relationship and collinearity analysis of P-type ATPase gene family in potato

**Figure 2:**
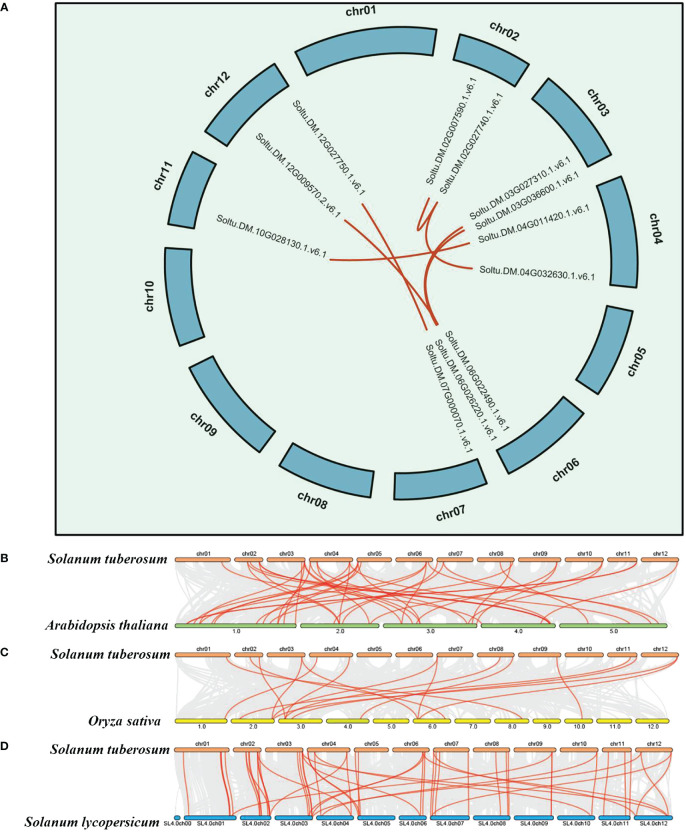
Syntenic and collinearity analysis of potato P-type ATPase gene family. **(A)** Green lines on the diagram represent potato chromosomes, while the deep red lines represent the putative orthologous P-type ATPase gene family of potato. **(B–D)** Collinearity analysis of P-type ATPase genes in potato, *Arabidopsis*, rice, and tomato. The chromosomes of *Arabidopsis*, tomato, rice, and potato are depicted in dark green, blue, yellow, and deep red, respectively. P-type ATPase genes exhibiting collinearity are illustrated by the deep red curve.

Gene duplication, tandem repeats, and whole-genome duplication are important factors driving the expansion of plant gene families. To elucidate the expansion and evolutionary characteristics of the P-type ATPase gene family in potatoes, we conducted whole-genome segmental duplication and collinearity analyses. Among all members of the gene family, only seven gene pairs (comprising 12 genes) were involved in the formation of segmental duplications (**Figure 2A**; **Supplementary Table S2**). The results indicate that tandem repeats of the P-type ATPase gene family mainly occur on chromosomes 7, 8, and 9. In particular, eight tandem gene family members on chromosome 9 formed a gene cluster (**Figure 2A**), which may have distinct functional characteristics compared to other family members. Collectively, these results indicate that both segmental and tandem duplications play roles in the expansion of the P-type ATPase gene family. As is well known, members of the same gene family in different species share a common ancestral origin, and collinearity analysis can effectively reveal the evolutionary relationships of specific gene families among different species. In the present study, we conducted a whole-genome collinearity analysis of potatoes with *Arabidopsis*, rice, and tomatoes (**Figures 2B-D**). These results indicate that compared to rice, potato and tomato genomes are highly syntonic, with more collinear gene pairs present. Additionally, many collinear gene pairs have been identified between potato and *Arabidopsis*, suggesting that the P-type ATPase gene family may have undergone a similar evolutionary history in potato, *Arabidopsis*, and tomato plants.

### 3.3 Conserved protein motifs, cis-regulatory element, and gene structure analysis of the P-type ATPase gene family

**Figure 3:**
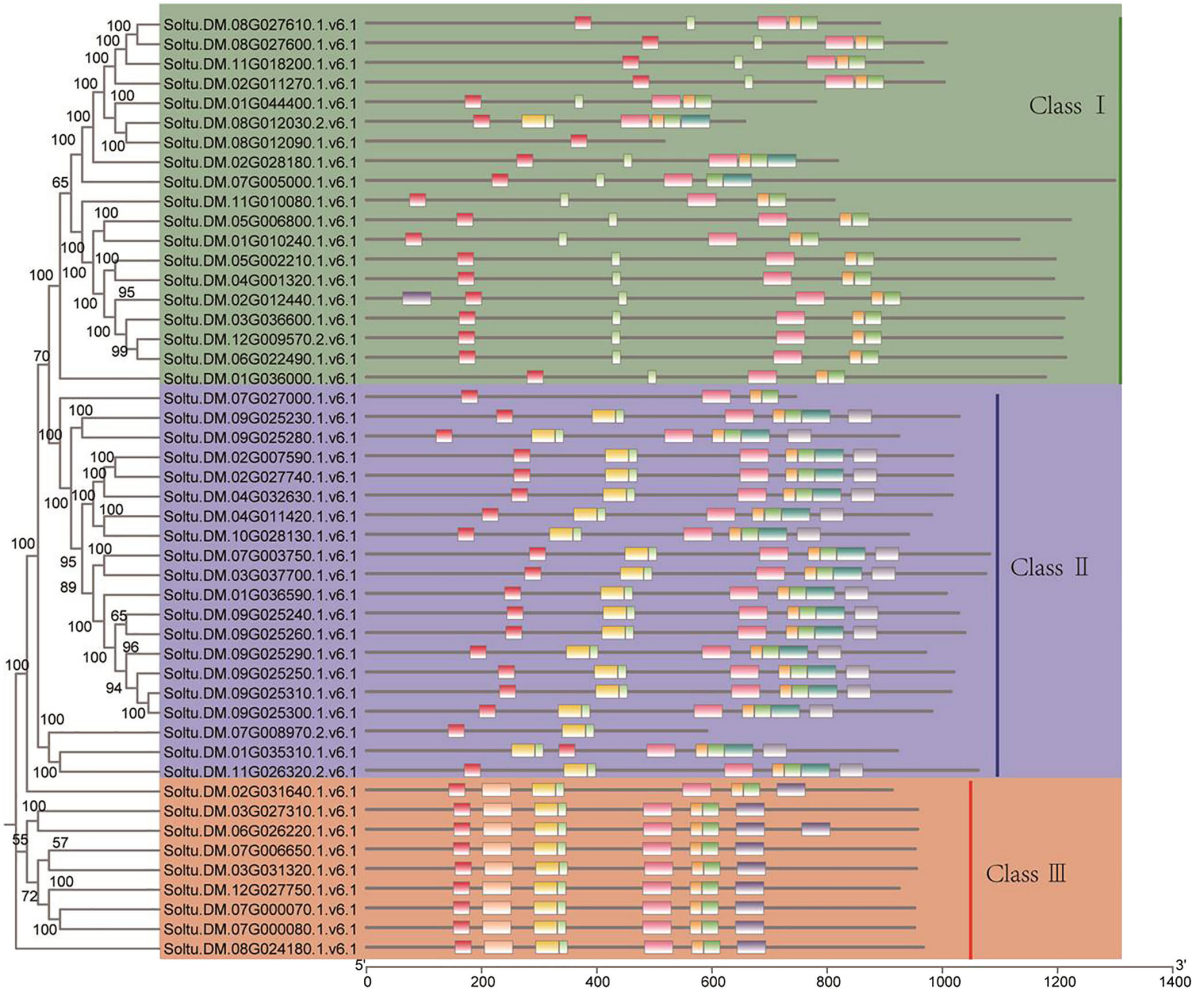
Evaluation of cis-regulatory element distribution within the promoters of potato P-type ATPase genes. Different colors indicate protein sequence motifs in P-type ATPase.

**Figure 4:**
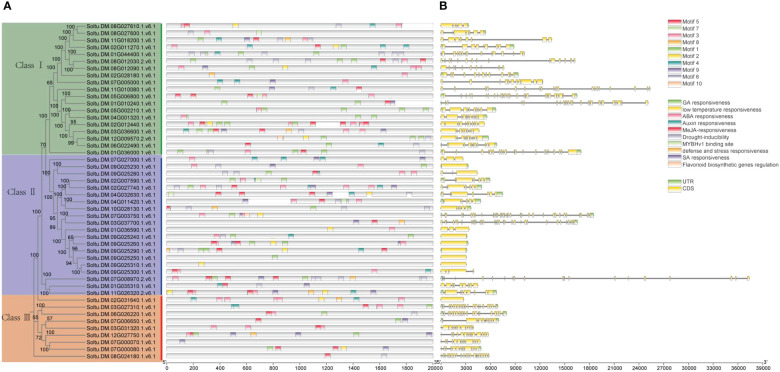
Assessment of the distribution of cis-regulatory elements within the promoters of potato P-type ATPase genes. **(A)** Distribution of cis-elements in various categories shown by the number. **(B)** Structural analysis reveals that different P-type ATPase genes contain fewer CDS regions.

A motif is a short sequence in a protein with specific functional or structural features that can affect its structure, stability, and activity. Motifs can also serve as signal sequences that guide protein localization and transport within cells. For the potato P-type ATPase gene family, we identified ten conserved motifs, with each protein sequence containing between one and eight motifs (**Figure 3**). For the potato P-type ATPase gene family, we identified 10 conserved motifs, with each protein sequence containing between one and eight motifs. Among these, some highly conserved motifs (such as motif 5) were identified in every protein sequence, whereas motifs 9 and 10 were found only in Class III protein sequences. Cis-acting elements in promoter regions regulate gene transcription. In addition to the identification of a large number of light-responsive elements, many hormone-related elements, such as ABA and GA response elements, were found in the promoter region of the P-type ATPase gene family (**Figure 4A**). Furthermore, many promoters contain cis-acting elements that respond to stress such as low temperature and drought, suggesting that members of the P-type ATPase gene family may play diverse functional roles in potatoes. Additionally, the results of the gene structure, phylogenetic tree, and protein motif analyses were highly consistent and all members of the P-type ATPase gene family were classified into three major classes. Overall, compared with Classes I and III, Class II genes contained fewer CDS, whereas Class I sequences contained many longer intron sequences than Class III (**Figure 4B**).

### 3.4 Phylogenetic and syntenic analysis of potato PM H^+^-ATPase proteins

**Figure 5:**
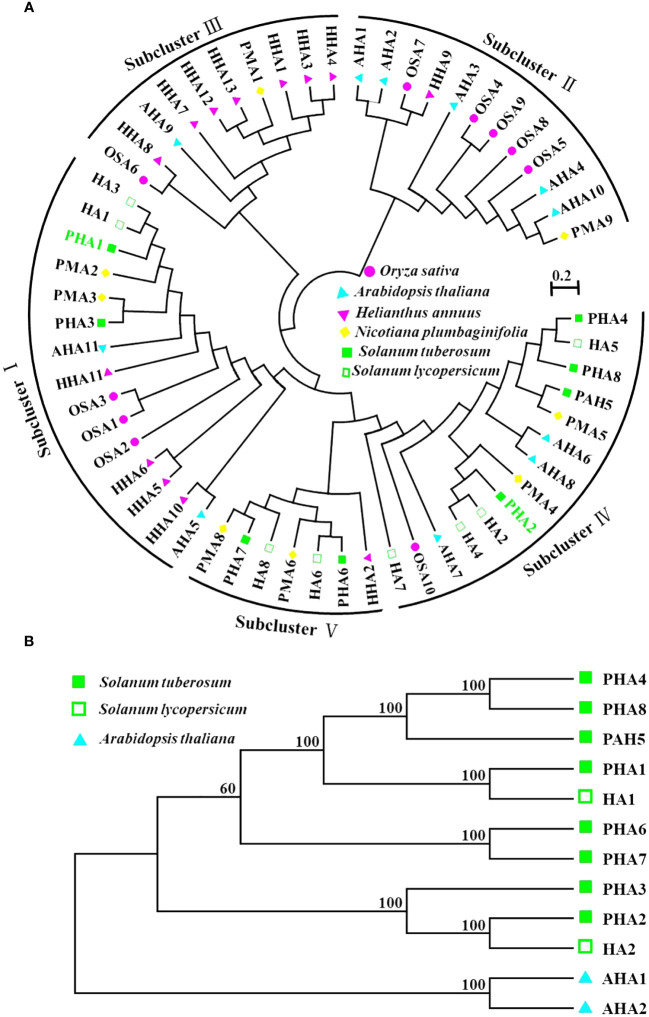
Phylogenetic analysis of PM H^+^-ATPase in potato. **(A)** Phylogenetic analysis of the complete protein sequences of PM H^+^-ATPase proteins from *Solanum tuberosum*, *Solanum lycopersicum*, *Arabidopsis thaliana*, *Oryza sativa*, *Helianthus annuus*, and *Nicotiana plumbaginifolia*. The neighbor-joining (NJ) tree was generated using the MEGA software, applying the pairwise deletion option, and 1,000 bootstrap replicates were performed to evaluate the reliability of the tree. In the generated tree, the PM H^+^-ATPase proteins from *Solanum tuberosum*, *Solanum lycopersicum*, *Arabidopsis thaliana*, *Oryza sativa*, *Helianthus annuus*, and *Nicotiana plumbaginifolia* are depicted as red triangles, green circles, and purple squares, respectively. **(B)** Evolutionary analysis of PM H^+^-ATPase (PHA) family proteins and HA1, HA2, AHA1, and AHA2 proteins.

As a superfamily of membrane proteins, P-type ATPases play important roles in plants and are involved in several fundamental physiological processes (Zhang et al., 2020). Particularly noteworthy are the PM H^+^-ATPase family members of the P3 subfamily, which play key roles in plant growth, development, and stress resistance (Morth et al., 2011; Falhof et al., 2016; Li et al., 2022). These enzymes maintain the stability of the intra- and extracellular environments by hydrolyzing ATP to drive ion transport and maintain electrochemical equilibrium across the membrane (Morth et al., 2011). In this study, we used 58 PM H^+^-ATPase protein sequences from six different species (*Solanum tuberosum*, *Solanum lycopersicum*, *Arabidopsis thaliana*, *Oryza sativa*, *Helianthus annuus*, and *Nicotiana plumbaginifolia*) to construct maximum likelihood trees to explore possible functional clustering relationships among PM H^+^-ATPase family member genes (**Figure 5**). The results showed that PM H^+^-ATPases could be classified into five subfamilies and PM H^+^-ATPase genes from species with close relatives tended to cluster. For example, PMA3 and PHA3 in sub-cluster I clustered with a bootstrap value of 100. AHA1, AHA1, OSA7, HHA9, AHA10, and PMA9 also formed a preferred cluster in Subcluster II. The PM H^+^-ATPase genes of *Helianthus annuus* were mainly clustered together in sub-cluster III. Interestingly, the PM H^+^-ATPase genes of potato and tomato showed closer affinity in the phylogenetic tree, whereas AHA7 formed a separate branch and was related to rice OSA10 and *Arabidopsis* AHA7. In conclusion, this study provides an in-depth analysis of the functional clustering relationships among PM H^+^-ATPase family member genes and lays the groundwork for further research on the functions of these genes.

### 3.5 Prediction and analysis of the spatial structure of PM H^+^-ATPase protein family members

**Figure 6:**
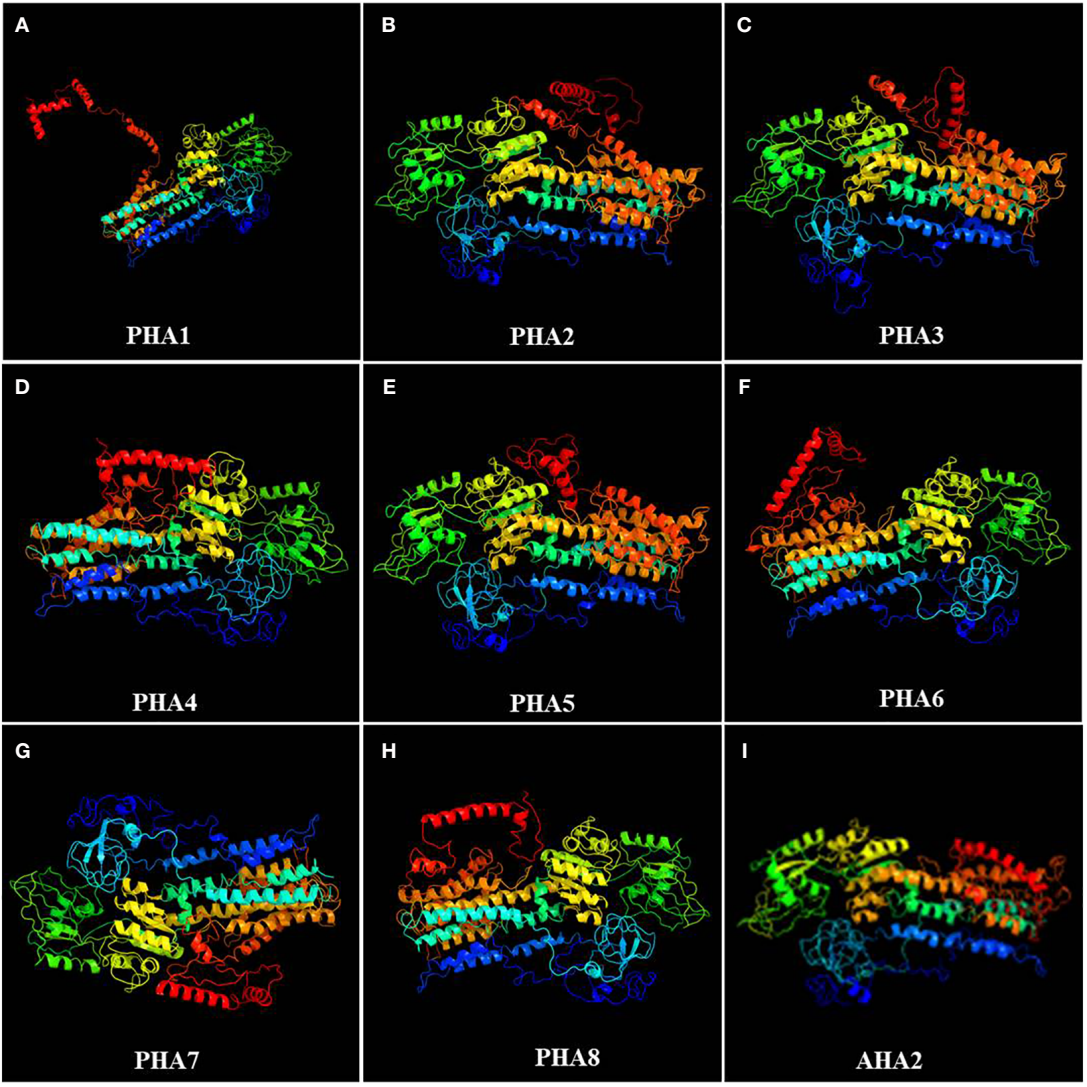
Tertiary structures of members of the PM H^+^-ATPase family: **(A)** PHA1, **(B)** PHA2, **(C)** PHA3, **(D)** PHA4, **(E)** PHA5, **(F)** PHA6, **(G)** PHA7, **(H)** PHA8, and **(I)** AHA2.

To further investigate potato PM H^+^-ATPase family proteins, their spatial structures were predicted using an online software. An analysis of the secondary structures of the PM H^+^-ATPase family proteins revealed that all proteins consist of three different structural units, including α-helices, random coils, and extended chains. However, the proportion and sequential distribution of these structural units vary among proteins. Random coils had the highest proportion, ranging from 50.22% to 55.24%, followed by α-helices, ranging from 35.95% to 39.43%. The proportion of extended chains varied less among different proteins, ranging from 8.10% to 10.71% (**Supplementary Figure S3**). Interestingly, among all PHA family proteins, PHA8 had the highest percentage of α-helical structures. PHA6, on the other hand, had the highest percentage of random coil structures and lowest percentage of extended strands, while the opposite was true for PHA5. Based on the publicly reported cryo-electron microscopy structure of AHA2, the tertiary structure of the PHA protein family was predicted using Phyre2 online software. The results showed that the tertiary structures of PHA2, PHA3, and PHA5 were similar to that of AHA2 in *Arabidopsis* (**Figure 6**). These findings provide important clues for exploring the function and mechanism of action of PM H^+^-ATPases and lay the foundation for a deeper understanding of the biological functions of the PM H^+^-ATPase protein family and their roles in resistance to late blight.

### 3.6 Expression variation of the PM H+-ATPase gene family in different organs of potato

**Figure 7:**
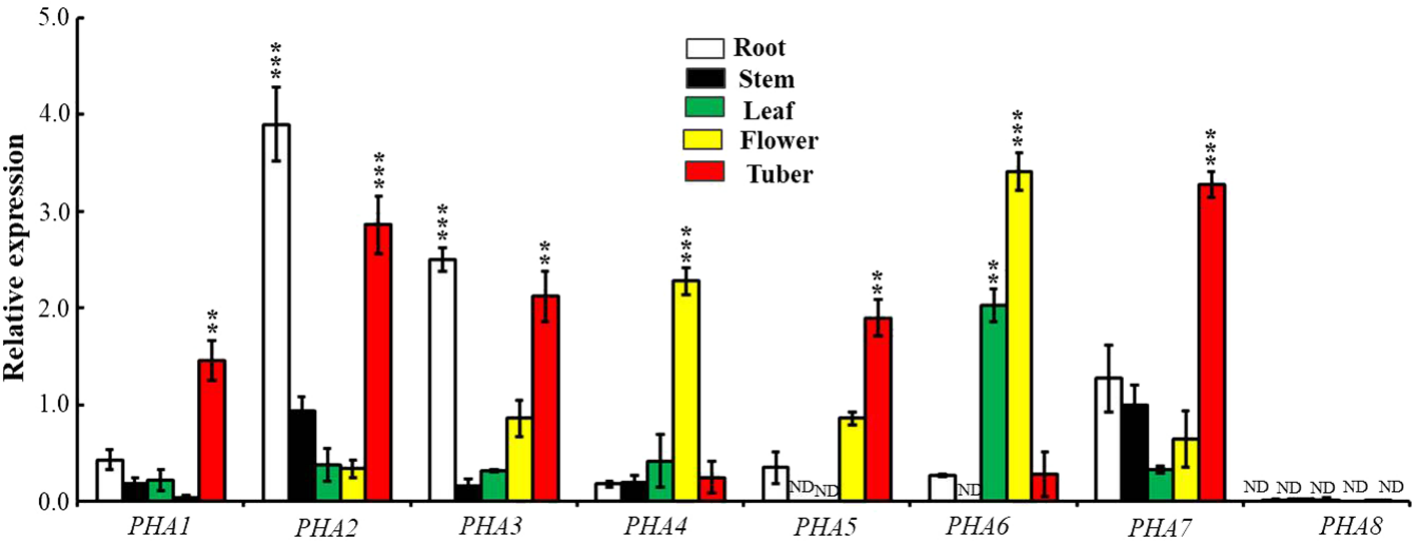
Expression patterns of PM H^+^-ATPase family members in various tissues. The expression levels of *PHA1*, *PHA2*, *PHA3*, *PHA4*, *PHA5*, *PHA6*, *PHA7* and *PHA8* were examined in a 7-week-old potato. To normalize the data, the expression of the *ACTIN* gene was used as an internal control. The statistical analysis was performed based on three independent replicate experiments and the results are presented as the mean ± standard deviation. Asterisks represent a significant difference between the gene and the actin under the same tissue (Student’s t-test; **, P<0.01; ***, P<0.001). "ND" stands for "not detected".

Examining the differences in the expression of *PHAs* in various organs provides insights into the functional roles of PM H^+^-ATPase family members in different organs. The expression levels of different *PHAs* vary across roots, stems, leaves, sprouts, and tubers of potatoes (**Figure 7**). In the roots and tubers, *PHA2*, *PHA3*, and *PHA7* exhibited the highest expression levels (10-fold, 7.8-fold, and 3.8-fold, respectively), followed by leaves. The flowers exhibited the lowest expression levels. In the leaves, *PHA6* showed relatively high expression levels, which were 7.5-fold higher than those in the roots, followed by the stems, where the expression levels were the lowest among the organs. Additionally, *PHA6* exhibited higher expression levels in flowers than in other organs but was lower overall than in other genes. In summary, there were differences in expression among PHA family members in different organs, with significantly higher expression of *PHA2* and *PHA3* in the roots and high expression of *PHA5* and *PHA6* in flower petals. Interestingly, there was no detectable expression of potato *PHA8* in any of the tissues, similar to the results in tomato (Liu et al., 2016). These findings provide significant clues for understanding the functional characteristics of *PHA* gene family members in different organs.

### 3.7 Expression analysis of PM H^+^-ATPase subfamily genes in potato in response to Pep13 treatment

**Figure 8:**
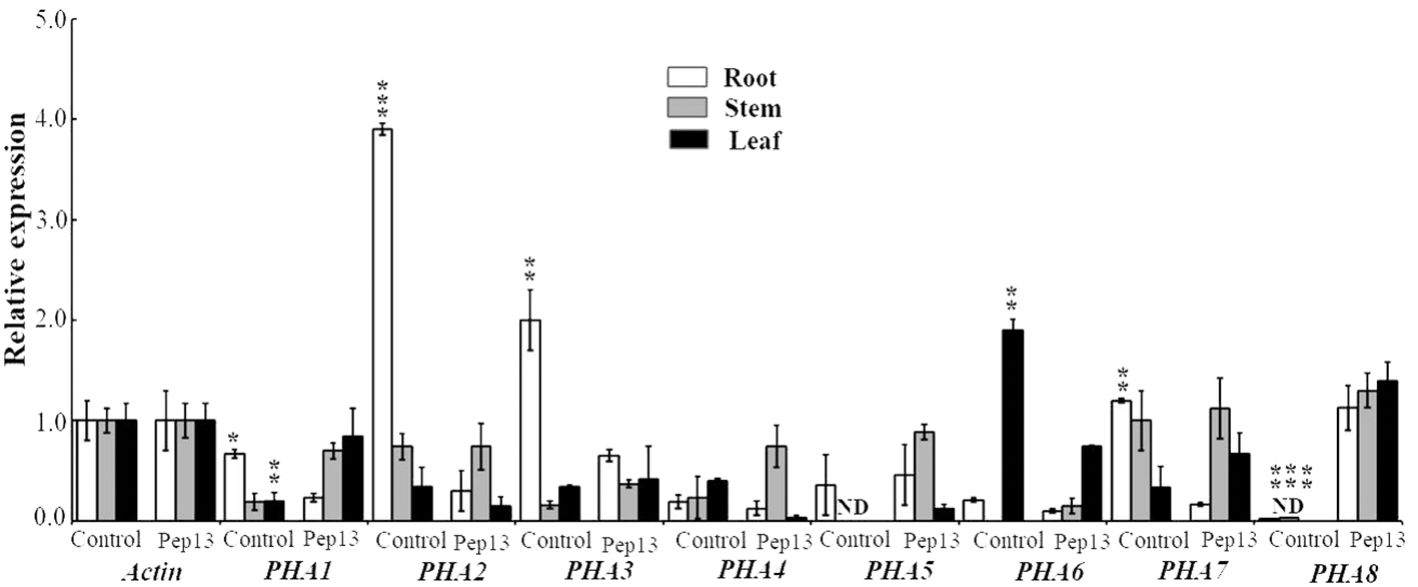
Expression levels of *PHA1*, *PHA2*, *PHA3*, *PHA4*, *PHA5*, *PHA6*, *PHA7*, and *PHA8* genes, which are associated with Pep13 in potato, after treatment. The internal standard used for normalization was *ACTIN*. Statistical analysis was performed and the data are presented as the mean ± standard deviation from three independent experiments. Asterisks represent a significant difference between the treatment group and the control under the same culture conditions (Student’s t-test; *, P<0.05; **, P<0.01; ***, P<0.001). "ND" stands for "not detected".

In this study, potatoes were treated with Pep13, and the expression of the PM H^+^-ATPase subfamily genes *PHA1*, *PHA2*, *PHA3*, *PHA4*, *PHA5*, *PHA6*, *PHA7*, and *PHA8* was analyzed in the roots, stems, and leaves (**Figure 8**). The results showed that the expression levels of *PHA1*, *PHA2*, *PHA3*, and *PHA7* decreased in Pep13-treated roots. Specifically, *PHA2* expression was significantly down-regulated after Pep13 treatment. However, in the leaves, only *PHA1* showed some degree of up-regulation. In contrast, the expression levels of seven of the eight genes remained unchanged in the stems. The highest expression level of the *PHA8* gene was found in the Pep13-treated potato roots, stems, and leaves. This suggests that Pep13 treatment may affect extracellular alkalinization in potato roots and modulate resistance to late blight pathogens by regulating the expression of the *PHA2* gene. Notably, *PHA8* was expressed only under Pep13 treatment conditions and its expression could not be detected in the absence of stress. This finding is similar to that previously reported for tomatoes (Liu et al., 2016, 2020). Taken together, these findings indicate that Pep13 treatment significantly affects the expression levels of the PM H^+^-ATPase subfamily genes *PHA1*, *PHA2*, *PHA3*, and *PHA8* in potato roots and leaves. These findings provide important clues for further studies on the mechanisms underlying the role of PM H^+^-ATPases in potato late blight resistance.

### 3.8 Role of the PHA2 gene in potato resistance to Pep13 treatment

**Figure 9:**
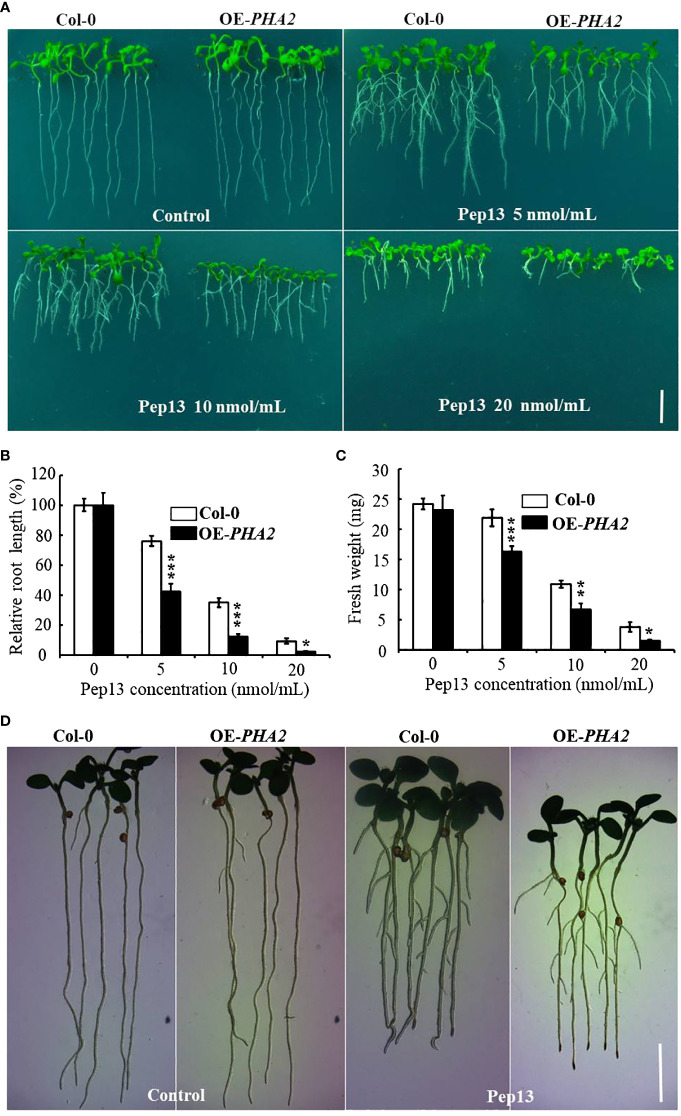
Growth phenotype of Col-0 and OE-*PHA2* under Pep13 treatment: **(A)** growth phenotype of Col-0 and OE-*PHA2* was observed after treatment with different concentrations of Pep13 (0, 5, 10, and 20 nM); **(B)** statistical analysis of relative root length; **(C)** statistical analysis of fresh weight. Images were captured on the 9th day of cultivation, and a scale bar of 1.0 cm was used. Statistical data represent the mean ± standard deviation of three independent experiments. Asterisks represent a significant difference between the treatment group and the control under the same culture conditions (Student’s t-test; *, P<0.05; **, P<0.01; ***, P<0.001). **(D)** Medium acidification surrounding root structures. Seedlings that were seven days old and germinated on 1/2 MS medium were subsequently moved to 1/2 MS or Pep13 medium for a total of 12 h The Pep13 medium contained 0.003% bromocresol purple and had a pH of 5.8.

To investigate the role of *PHA2* in the development of potato late blight, we conducted experiments using wild-type (Col-0) and heterologously overexpressing plants (OE-*PHA2*) plants treated with different concentrations of Pep13 (0, 5, 10, and 20 nM). The results revealed no significant differences in the growth phenotypes between Col-0 and OE-*PHA2* without Pep13 treatment (**Figure 9**). However, upon treatment with Pep13, the growth phenotype of OE-*PHA2* changed significantly. In particular, root growth was severely inhibited and the degree of inhibition increased at higher Pep13 concentrations (**Figure 9A**). In contrast, Col-0 exhibited better growth after Pep13 treatment, with less severe inhibition of root growth than OE-*PHA2.* Biomass relative to root length and fresh weight displayed similar trends (**Figures 9B, C**). These findings suggest that the overexpression of *PHA2* weakens *Arabidopsis* resistance to Pep13 treatment, consequently reducing its resistance to late blight pathogens. Interestingly, *AHA2* activity in *Arabidopsis* has been detected using a pH dye (bromocresol violet) (Wang et al., 2022). The results demonstrated that acidification near the roots was affected by Pep13-treated conditions in both Col-0 and OE-*PHA2* plants but the acidification levels were higher in OE-*PHA2* plants than in Col-0 plants (**Figure 9D**). These observations indicated that *PHA2* plays a crucial role in late blight development and may be involved in the regulation of plant resistance to pathogens.

### 3.9 Relationship between PM H^+^-ATPase enzymatic activity in the potato and Pep13

**Figure 10:**
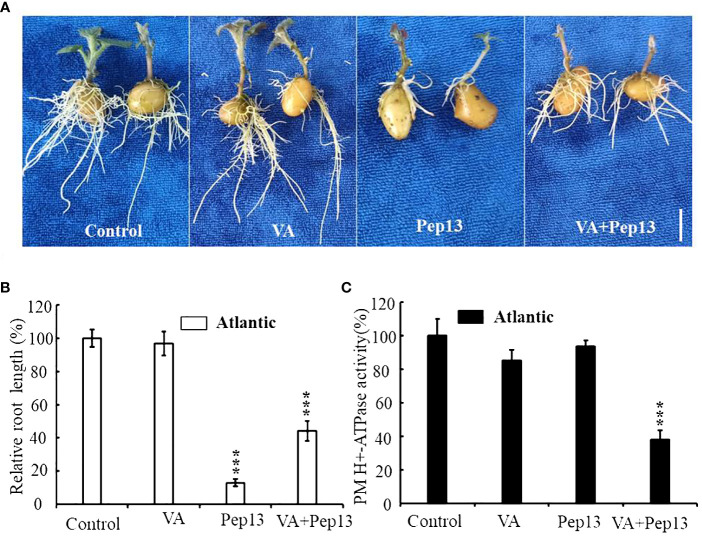
Resistance of the potato cultivar Atlantic to external vanadate (VA), Pep13, and their combination. **(A)** Growth phenotype of the Atlantic potato cultivar under VA, Pep13, and VA and Pep13 treatment; **(B)** Relative root length statistics; pictures were taken on the 12^th^ day after cultivating. Scale bar = 1.0 cm. **(C)** PM H^+^-ATPase activity was measured in microsomal membranes isolated from 3-week-old wild-type (WT) roots. The roots were treated with VA, Pep13, and VA and Pep13 for 72 h The PM H^+^-ATPase activity in the control condition was set as 1.0. The data presented are the mean ± SD of three independent experiments. Asterisks represent a significant difference between the treatment group and the control under the same culture conditions (Student’s t-test; ***, P<0.001).

To investigate the effect of Pep13 on the enzymatic activity of potato PM H^+^-ATPase, a series of experiments were conducted. The medium was supplemented with VA, Pep13, and their combination and the growth of Atlantic potato roots was observed. The results demonstrated that Pep13 significantly inhibited the growth of potato roots. However, when VA was added, the extent of the inhibition caused by Pep13 was alleviated (**Figure 10A**). Analysis of relative root length supported these findings (**Figure 10B**). To further investigate the enzymatic activity of PM H^+^-ATPase in the root system, PM H^+^-ATPase was extracted from Atlantic potato roots subjected to various treatments. Enzyme activity was then measured. After 3 days of treatment with 10 µM VA, PM H^+^-ATPase activity in potato Atlantic roots was inhibited by approximately 85% compared to the control (**Figure 10C**). Interestingly, PM H^+^-ATPase activity in potato Atlantic roots was lower when both VA and Pep13 were present than when only Pep13 was present (**Figure 10C**). These observations suggest that invasion by potato late-blight pathogens can inhibit the activity of PM H^+^-ATPase, which in turn leads to extracellular alkalinization as a signaling molecule. This signaling molecule regulates processes such as growth inhibition, increased immunity, and improved environmental adaptation.

## 4 Discussion

Previous studies have identified and analyzed P-type ATPase gene family members in different plants. The number and composition of the P-type ATPase family members vary among different plants, indicating the diversity and complexity of these proteins. For instance, in *Arabidopsis thaliana*, a total of 46 P-type ATPase gene family members have been identified (Palmgren, 2001). Similarly, in the rice genome, which is over three times the size of that of *Arabidopsis*, 43 P-type ATPase gene family members have been identified (Baxter et al., 2003). In this study, we characterized the potato P-type ATPase gene and identified 48 members distributed across 12 different chromosomes (**Figure 1**). These findings are consistent with previous studies on the chromosomal distribution of P-type ATPase genes in other species. To further classify the subfamilies of the potato P-type ATPase genes, we constructed a phylogenetic tree using the protein sequences of these 48 genes (**Supplementary Figure S1**). The results revealed that potato P-type ATPase genes could be categorized into five subfamilies: P1B, P2A, P2B, P3A, and P4. Among these subfamilies, the number of genes in the P4 subfamily was relatively low, whereas the number of genes in other subfamilies was higher. Interestingly, within the P3A subfamily, among the 12 P-type ATPase gene members, eight genes identified as PM H^+^-ATPases clustered. These findings provide valuable insights into P-type ATPase genes in potatoes.

To study the potato P-type ATPase gene family, we focused on the PM H^+^-ATPase genes of the P3 subfamily (**Supplementary Table S3**). Previous studies have shown that PM H^+^-ATPase genes have diverse functions, particularly in disease resistance, in different plant species (Ortiz-Morea et al., 2016; Liu et al., 2022). To gain a deeper understanding of the functions and regulatory mechanisms of this gene family, we analyzed the expression of the PM H^+^-ATPase gene family in different potato tissues (**Figure 7**). The results showed significant differences in the expression levels of the PM H^+^-ATPase gene family in the potato root, stem, and leaf tissues. Interestingly, *PAH8* was only expressed under pathogen stress and could not be detected in normal potato tissues, which is consistent with previous studies on tomatoes. RT-qPCR assays revealed that *PHA2* had a positive correlation with cytoplasmic membrane acidification after treatment with the small peptide Pep13, as reported in previous studies. This finding further supports the important role of PM H^+^-ATPases in the regulation of cellular acid-base homeostasis. These findings are significant for understanding the regulation of cellular acid-base homeostasis in potatoes and for improving their resistance to pathogens, such as late blight. They also provide useful references for further investigations of the functions and applications of the P-type ATPase gene family.

PM H^+^-ATPase is a key proton pump in the plasma membrane of plant cells and its main function is to establish a proton electrochemical gradient across the plasma membrane (Sondergaard et al., 2004). Recent studies have shown that PM H^+^-ATPases play important regulatory roles in the immune response of plants (Schaller and Oecking, 1999; Lee et al., 2022). In *Arabidopsis*, for example, the signaling molecule Pep1 inhibits root growth by interfering with acid-base signaling. Interestingly, the inhibitory effect of Pep1 on root growth gradually diminishes with increasing environmental acidity, whereas under neutral and alkaline conditions, the inhibitory effect of Pep1 on root growth is diminished (Shen et al., 2020). This suggests that the acidity or alkalinity of the environment has an important effect on the activity of Pep1. However, in this study, we discovered that the small peptide Pep13 inhibited potato PM H^+^-ATPase, limited proton efflux to the outer layer of root cells, and enhanced disease resistance. This finding triggered an in-depth study to determine whether Pep13 inhibits PM H^+^-ATPase activity, which plays an important role in regulating root immunity.

Previous studies have shown that flg22 and RAPID ALKALINIZATION FACTORs (RALFs) induce extracellular alkalinization by inhibiting PM H^+^-ATPase activity (Benschop et al., 2007; Haruta et al., 2014). Pep1 signaling is believed to amplify flg22 signaling in plant leaves (Bartels et al., 2013; Zheng et al., 2018), whereas the two exhibit different effects on the regulation of PM H^+^-ATPase activity in roots. This raises the need for further consideration of whether Pep13 inhibited PM H^+^-ATPase activity plays a crucial role in regulating root immunity, requiring further investigation. Therefore, a comprehensive study on the interaction between Pep13 and PM H^+^-ATPase is essential to fully understand the intricacies of plant immunomodulatory mechanisms. This study aimed to uncover novel regulators of plant root immune responses and establish a crucial theoretical foundation for the development of new plant immune enhancement strategies. However, additional experimental studies and data support are necessary to fully comprehend the specific effects of Pep13-inhibited PM H^+^-ATPase activity on root immune regulation. In future studies, we plan to utilize CRISPR/Cas9 technology to generate single and double mutants of the *StPHA2* and *StPEPR1*. This research is of significant value as it aims to further confirm the critical role of the *StPHA2* gene in enabling potatoes to combat the invasion of late blight pathogens from a molecular genetics perspective. Simultaneously, it will also validate the importance of Pep13.

In conclusion, we conducted a genome-wide analysis and identified 48 P-type ATPase genes. These genes were categorized into five subgroups and found to be unevenly distributed across the 12 chromosomes. RT-qPCR revealed that all PM H^+^-ATPase genes except *PHA8* were expressed in at least one tissue. Specific expression was mainly observed in leaves, roots, stems, tubers, and flowers. The RT-qPCR results indicated that *PHA1*, *PHA2*, *PHA3*, and *PHA7* were sensitive and quickly down-regulated when exposed to Pep13, suggesting their important role in potato resistance to late blight. To investigate this further, transgenic *Arabidopsis* was generated by introducing *PHA2* into wild-type *Arabidopsis thaliana* using the Agrobacterium inflorescence infestation method. Phenotypic and resistance analyses demonstrated that overexpression of *PHA2* affected *Arabidopsis* root resistance to the small peptide Pep13, resulting in a root growth-sensitive phenotype. Additionally, the overexpression of *PHA2* increased the PM H^+^-ATPase activity of *Arabidopsis* strains to some extent. This study explored the genetic resources of potatoes in response to late blight, elucidated the functional role of *PHA2* in late blight virulence, provided a theoretical foundation for understanding the molecular immune mechanism of potato resistance to late blight, and identified important genetic resources for the selection and breeding of new potato varieties with high resistance to late blight.

## Data availability statement

The original contributions presented in the study are included in the article/**Supplementary Material**. Further inquiries can be directed to the corresponding author.

## Author contributions

YA: Data curation, Formal analysis, Writing – review & editing. FZ: Writing – original draft, Writing – review & editing, Formal analysis, Investigation, Methodology. AY: Formal analysis, Writing – original draft. ZN: Formal analysis, Resources, Writing – original draft. MC: Formal analysis, Funding acquisition, Methodology, Supervision, Writing – review & editing.

## Supplementary material

Supplementary Figure 1 RT-PCR identification of transgenic lines overexpressing the *PHA2* gene.

Supplementary Figure 2 Phylogenetic tree of the potato P-type ATPase family, which can be divided into five distinct subfamilies: P1B, P2A, P2B, P3A, and P4. The neighbor-joining (NJ) tree was constructed using the MEGA software with the pairwise deletion option, and 1,000 bootstrap replicates were used to assess tree reliability.

Supplementary Figure 3 The secondary structure characteristics of members within the PM H^+^-ATPase (PAH) family. **(A)** The composition of three protein secondary structure elements in each member of the PM H^+^-ATPase family was determined. **(B)** The distribution pattern of these three protein secondary structure elements was analyzed for each individual within the PM H^+^-ATPase family. In the corresponding figure, the alpha helix is represented by a blue line, the random coil by a purple line, and the extended chain by a red line.
