# Supplementary figures and images for: Identification of the potato (Solanum tuberosum L.) P-type ATPase gene family and investigating the role of PHA2 in response to Pep13
Source: Front Plant Sci. 2024 Jun 6;15:1353024. doi: 10.3389/fpls.2024.1353024 (PMC11187005; doi:10.3389/fpls.2024.1353024)

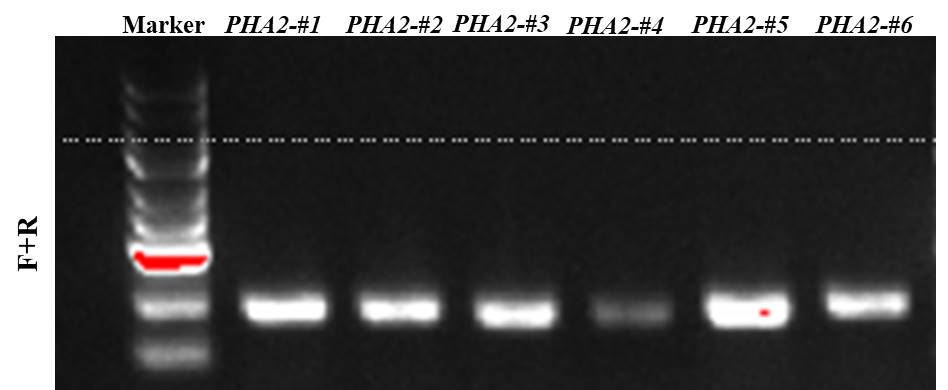

Supplement: Supplementary Figure 1 — RT-PCR identification of transgenic lines overexpressing the PHA2 gene. [file Image_1.jpeg]

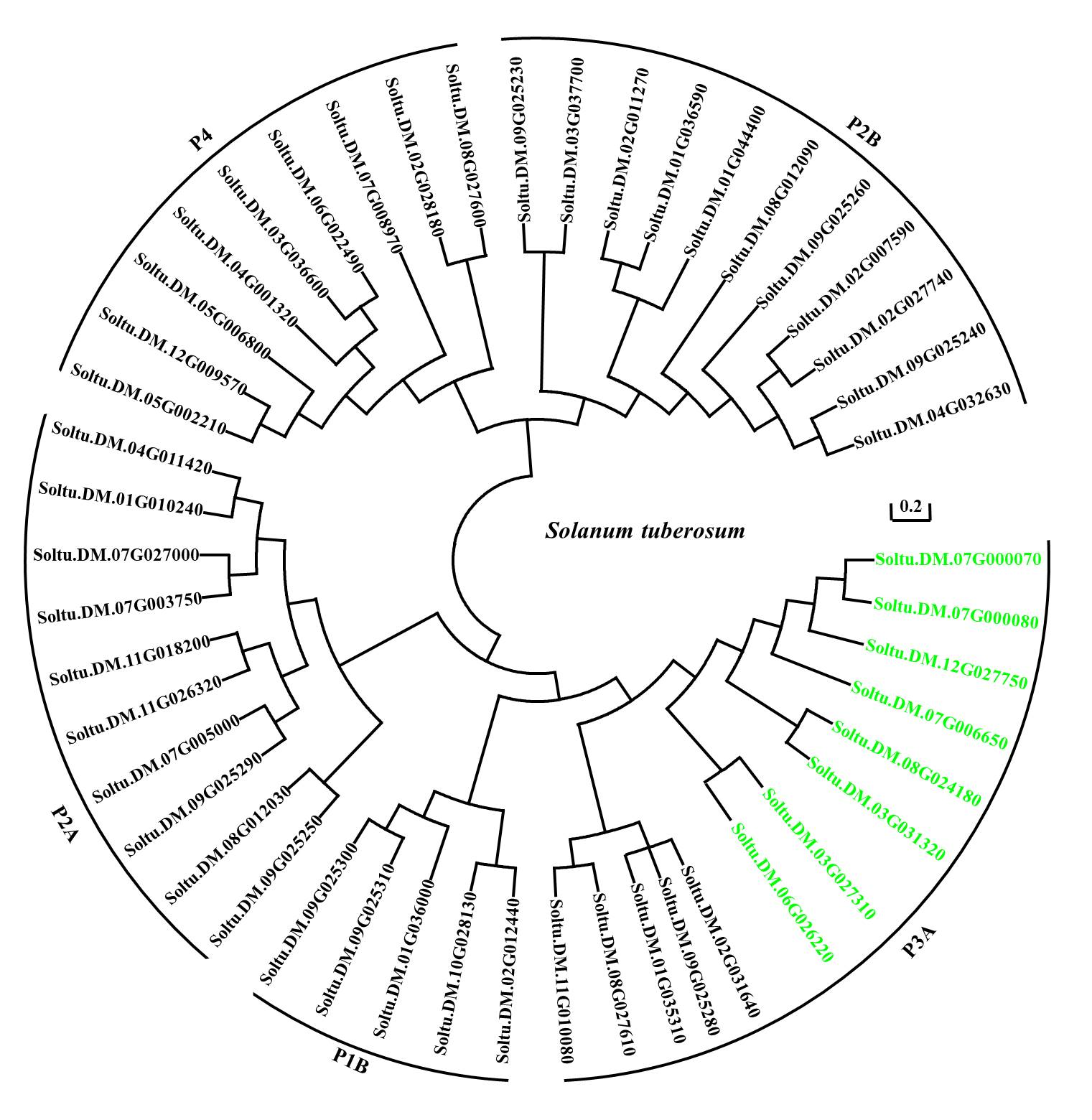

Supplement: Supplementary Figure 2 — Phylogenetic tree of the potato P-type ATPase family, which can be divided into five distinct subfamilies: P1B, P2A, P2B, P3A, and P4. The neighbor-joining (NJ) tree was constructed using the MEGA software with the pairwise deletion option, and 1,000 bootstrap replicates were used to assess tree reliability. [file Image_2.jpeg]

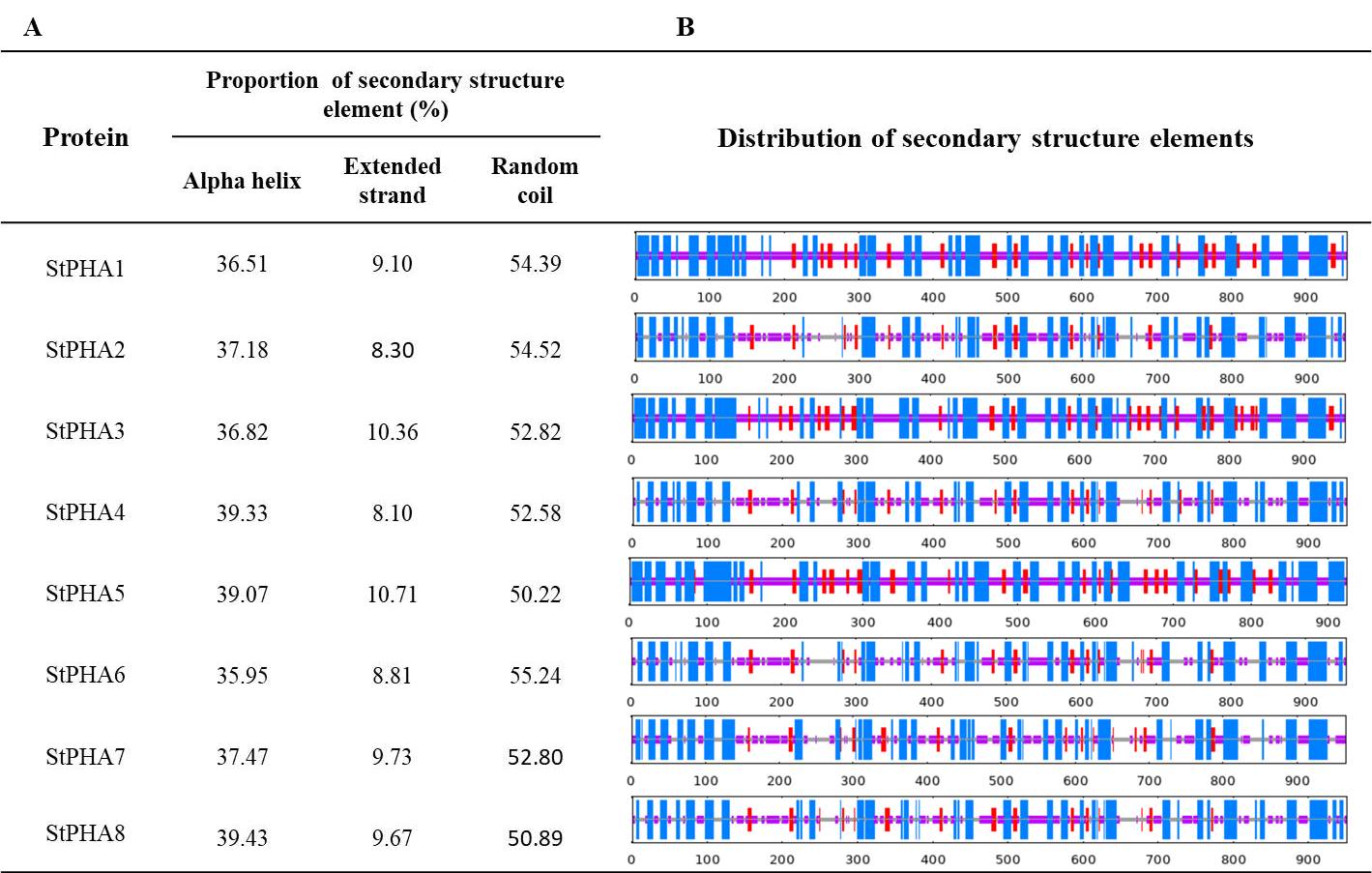

Supplement: Supplementary Figure 3 — The secondary structure characteristics of members within the PM H+-ATPase (PAH) family. (A) The composition of three protein secondary structure elements in each member of the PM H+-ATPase family was determined. (B) The distribution pattern of these three protein secondary structure elements was analyzed for each individual within the PM H+-ATPase family. In the corresponding figure, the alpha helix is represented by a blue line, the random coil by a purple line, and the extended chain by a red line. [file Image_3.jpeg]
