# Supplementary material for: Identification of the potato (Solanum tuberosum L.) P-type ATPase gene family and investigating the role of PHA2 in response to Pep13
Source: Front Plant Sci. 2024 Jun 6;15:1353024. doi: 10.3389/fpls.2024.1353024 (PMC11187005; doi:10.3389/fpls.2024.1353024)
Supplement: Supplementary file 4 [file Table_1.docx]

**Table S1.** Primers used in this study

| **Primers** | **Sequence 5′-3′** | **Purpose** |
| --- | --- | --- |
| Actin-F | GGGATGGAGAAGTTTGGTGGTGG | RT-qPCR |
| Actin-R | CTTCGACCAAGGGATGGTGTAGC |  |
| PHA1-F | CGTCTCGCTCCTAAAGCCAA |  |
| PHA 1-R | ACGAGCATCTGCAGGAACAA |  |
| PHA 2-F | AATGTTCGCGAGAGGGTCTG |  |
| PHA 2-R | CCAATCTGGGGGCTTTCCAT |  |
| PHA 3-F | AAGTTCCTCATCCGTTATGCT |  |
| PHA3-R | AAGCTCGCGCAGTTCTTTTC |  |
| PHA 4-F | TTTCGGCTTGAGTGGAAGGG |  |
| PHA 4-R | TGTCGTTGAATAGGCCAGCG |  |
| PHA 5-F | TGGGATCATCCTCGGAACCT |  |
| PHA 5-R | AAGACCGTGTTCGTTCTCCC |  |
| PHA 6-F | CGGAGTCTTTCCAGAGCACA |  |
| PHA 6-R | TATCACACTCAATCCTGGCTCC |  |
| PHA 7-F | AGATGCGACCAAGGACACTG |  |
| PHA 7-R | AACCTCTTGGATGCCTGCTC |  |
| PHA 8-F | TGGAAAGGCTGCTCATCTCG |  |
| PHA 8-R | ACAGCTTCACGAGGATGTCG |  |
| PAH2-F1 | AAAGCTATAAGCCTCGAAGAGATAA | Vector construction |
| PHA2-R1 | TGCTGCTGGATCGTTTCG |  |
| PAH-F2 | A AGAAAAGAAGAGACACACAAGC | Identification of heterologous overexpression |
| PHA-R2 | GACACAATCCCTTTCAATGG |  |
