# Supplementary material for: Identification of the potato (Solanum tuberosum L.) P-type ATPase gene family and investigating the role of PHA2 in response to Pep13
Source: Front Plant Sci. 2024 Jun 6;15:1353024. doi: 10.3389/fpls.2024.1353024 (PMC11187005; doi:10.3389/fpls.2024.1353024)
Supplement: Supplementary file 5 [file Table_2.docx]

**Table S2.** The seven pairs of genes (including 12 genes) involved in segmental duplication.

| **Pairing number** | | **Gene ID** | **Gene ID** | |
| --- | --- | --- | --- | --- |
| 1 | Soltu.DM.02G007590.1.v6.1 | | | Soltu.DM.02G027740.1.v6.1 |
| 2 | Soltu.DM.02G027740.1.v6.1 | | | Soltu.DM.04G032630.1.v6.1 |
| 3 | Soltu.DM.07G000070.1.v6.1 | | | Soltu.DM.12G027750.1.v6.1 |
| 4 | Soltu.DM.03G036600.1.v6.1 | | | Soltu.DM.06G022490.1.v6.1 |
| 5 | Soltu.DM.03G027310.1.v6.1 | | | Soltu.DM.06G026220.1.v6.1 |
| 6 | Soltu.DM.04G011420.1.v6.1 | | | Soltu.DM.10G028130.1.v6.1 |
| 7 | Soltu.DM.06G022490.1.v6.1 | | | Soltu.DM.12G009570.2.v6.1 |
