# Supplementary material for: Identification of the potato (Solanum tuberosum L.) P-type ATPase gene family and investigating the role of PHA2 in response to Pep13
Source: Front Plant Sci. 2024 Jun 6;15:1353024. doi: 10.3389/fpls.2024.1353024 (PMC11187005; doi:10.3389/fpls.2024.1353024)
Supplement: Supplementary file 6 [file Table_3.docx]

**Table S3** Molecular characteristics of *PHA* genes in potato.

| Gene ID | Gene name | | N-amino acids | | | Molecular weight | Theoretical pI | | Transmembrane domain | Subcellular localization |  |
| --- | --- | --- | --- | --- | --- | --- | --- | --- | --- | --- | --- |
| Soltu.DM.06G026220 | | *PHA1* | | 956 | 105040.91 | | | 6.31 | 8 | plas:12,cyto:1,vacu:1 | |
| Soltu.DM.07G006650 | | *PHA2* | | 952 | 105013.91 | | | 6.31 | 10 | plas:12,cyto:1,vacu:1 | |
| Soltu.DM.03G027310 | | *PHA3* | | 956 | 105117.26 | | | 6.31 | 8 | plas:12,cyto:1,vacu:1 | |
| Soltu.DM.07G000080 | | *PHA4* | | 951 | 104466.05 | | | 5.71 | 8 | Plas13, vacu1 | |
| Soltu.DM.12G027750 | | *PHA5* | | 924 | 101582.49 | | | 5.63 | 8 | plas:13, E.R.：1 | |
| Soltu.DM.03G031320 | | *PHA6* | | 954 | 105293.18 | | | 6.37 | 11 | plas:11,vacu:2,cyto:1 | |
| Soltu.DM.08G024180 | | *PHA7* | | 966 | 106154.21 | | | 6.79 | 8 | plas:12,vacu:1 | |
| Soltu.DM.07G000070 | | *PHA8* | | 951 | 104512.12 | | | 5.83 | 8 | plas: 13, vacu: 1 |  |
